# Effect of hepatic arterial dexamethasone administration on long-term survival among hepatocellular carcinoma patients undergoing transcatheter arterial chemoembolization

**DOI:** 10.1186/s12871-026-04054-w

**Published:** 2026-06-25

**Authors:** Fang Yan, Yan Wang, Yang Huang, Wei Xing, Ying Guo

**Affiliations:** 1 Department of Anesthesiology, State Key Laboratory of Oncology in South China, Guangdong Provincial Clinical Research Center for Cancer, Sun Yat-sen University Cancer Center, Guangzhou, 510060 P. R. China; 2 Department of Clinical Research, State Key Laboratory of Oncology in South China, Guangdong Provincial Clinical Research Center for Cancer, Sun Yat-sen University Cancer Center, Guangzhou, 510060 P. R. China

**Keywords:** Hepatic arterial dexamethasone, Hepatocellular carcinoma, Survival, Propensity score analyses, TACE

## Abstract

**Background:**

In cancer patients, dexamethasone has been linked to long-term survival outcomes, but there is uncertainty as to its effect on survival outcomes among patients with hepatocellular carcinoma (HCC) who undergo transcatheter arterial chemoembolization (TACE).

**Methods:**

We retrospectively reviewed HCC patients who underwent TACE as the initial treatment between January 2014 and December 2016. Patients were categorized into the dexamethasone group and no dexamethasone group. We conducted propensity score matching (PSM), inverse probability weighting (IPTW) and adjusted for propensity score. Our endpoints were progression-free survival (PFS) and overall survival (OS). We also performed exploratory analyses in subgroups to assess the association between hepatic arterial dexamethasone and survival outcomes in pre-specified subgroups.

**Results:**

Hepatic arterial dexamethasone was correlated with a favorable long-term survival in unadjusted and multivariate analysis. IPTW showed patients receiving hepatic arterial dexamethasone had prolonged PFS (HR = 0.69, 95%CI 0.49–0.95, *P* = 0.024) and OS (HR = 0.72, 95%CI 0.52-1.00, *P* = 0.047). Similar trends were observed in PSM and another propensity score analysis. Survival benefit of hepatic arterial dexamethasone remained in the subgroup undergoing platinum-based TACE.

**Conclusion:**

Hepatic arterial dexamethasone might be associated with better long-term survival in HCC patients undergoing TACE. This study highlights the clinical significance of hepatic arterial dexamethasone in HCC patients undergoing TACE.

**Supplementary Information:**

The online version contains supplementary material available at 10.1186/s12871-026-04054-w.

## Background

Hepatocellular carcinoma (HCC) ranks sixth among the most prevalent cancers and is the third most common cause of cancer mortality globally [1, 2]. More than 70% of HCC patients are diagnosed at an intermediate or advanced stage, surgical treatments are no longer suitable. Transcatheter arterial chemoembolization (TACE) is the standard first-line therapy for HCC with intermediate or advanced stage [3, 4]. However, the treatment outcome of these HCC patients remains poor, 5-year survival rate remains less than 20% [5]. Undoubtedly, efforts should urgently be made by finding therapies that can improve patient survival.

Dexamethasone is commonly used as adjuvant medication during TACE [6, 7]. Post-TACE inflammatory response and adverse reactions of chemotherapeutics may contribute to the incidence of postembolization syndrome (PES), which is characterized by fever, abdominal pain, nausea and vomiting [8]. A previous clinical trial has shown that dexamethasone can reduce the incidence and severity of TACE-related PES, possibly by suppressing inflammation [9]. Also, it has gained more attention due to its direct preventive role in cancer therapy. Previous study has reported that dexamethasone could prevent hepatocellular carcinogenesis via the induction of a glycolysis-to-gluconeogenesis switch in mice [10]. Similar laboratory studies have shown that glucocorticoid might attenuate early progression of HCC through the inhibition of paraneoplastic inflammation and angiogenic pathways [11]. 

Besides, dexamethasone, used as a chemosensitizer and chemoprotectant in cancer chemotherapy, may play a crucial role in indirect antitumor effects. A recent study has revealed that dexamethasone might enhance cisplatin chemosensitivity of HCC cells by modulating tumor angiogenesis and cell cycle kinetics [12]. Another study has demonstrated that dexamethasone could increase the therapeutic sensitivity of cancer cells via inhibiting SUMOylation of Oct4 and HIF‑1α [13]. In vivo rat models of colorectal hepatic metastasis, hepatic arterial dexamethasone infusion was implicated in an elevated tumor response rate and a trend toward improved survival [14]. Moreover, a randomized controlled trial indicated that hepatic arterial dexamethasone infusion was associated with increased chemosensitivity and a trend toward longer survival in patients with colorectal hepatic metastasis [15]. However, it is uncertain whether hepatic arterial dexamethasone provides a long-term survival benefit for HCC patients who receive TACE.

Therefore, we performed a retrospective propensity score analysis to explore the impact of hepatic arterial dexamethasone on long-term survival in HCC patients who undergo TACE. We further investigate whether combining hepatic arterial dexamethasone with platinum-based chemotherapy confers a survival benefit in HCC patients who undergo TACE.

## Methods

The retrospective cohort study followed the strengthening the reporting of observational studies in epidemiology (STROBE) reporting guideline. This study was approved by the Institutional Review Board (IRB) at Sun Yat-Sen University Cancer Center (SYSUCC), the largest cancer center in southern China (approval no. B2025-127-01). Considering the retrospective design of this study, written informed consent was waived. This study was performed in accordance with the Declaration of Helsinki.

### Patient selection and data collection

We performed a retrospective cohort study of consecutive HCC patients who received TACE as first-line therapy from a prospectively maintained single-institutional database between January 2014 and December 2016. The diagnosis of hepatocellular carcinoma was made pathologically or clinically according to the criteria established by the American Association for the Study of Liver Diseases and the European Association for the Study of the Liver. Eligibility for inclusion required: HCC patients treated by TACE as first-line therapy and patients aged 18 to 75 years.

**Fig. 1:**
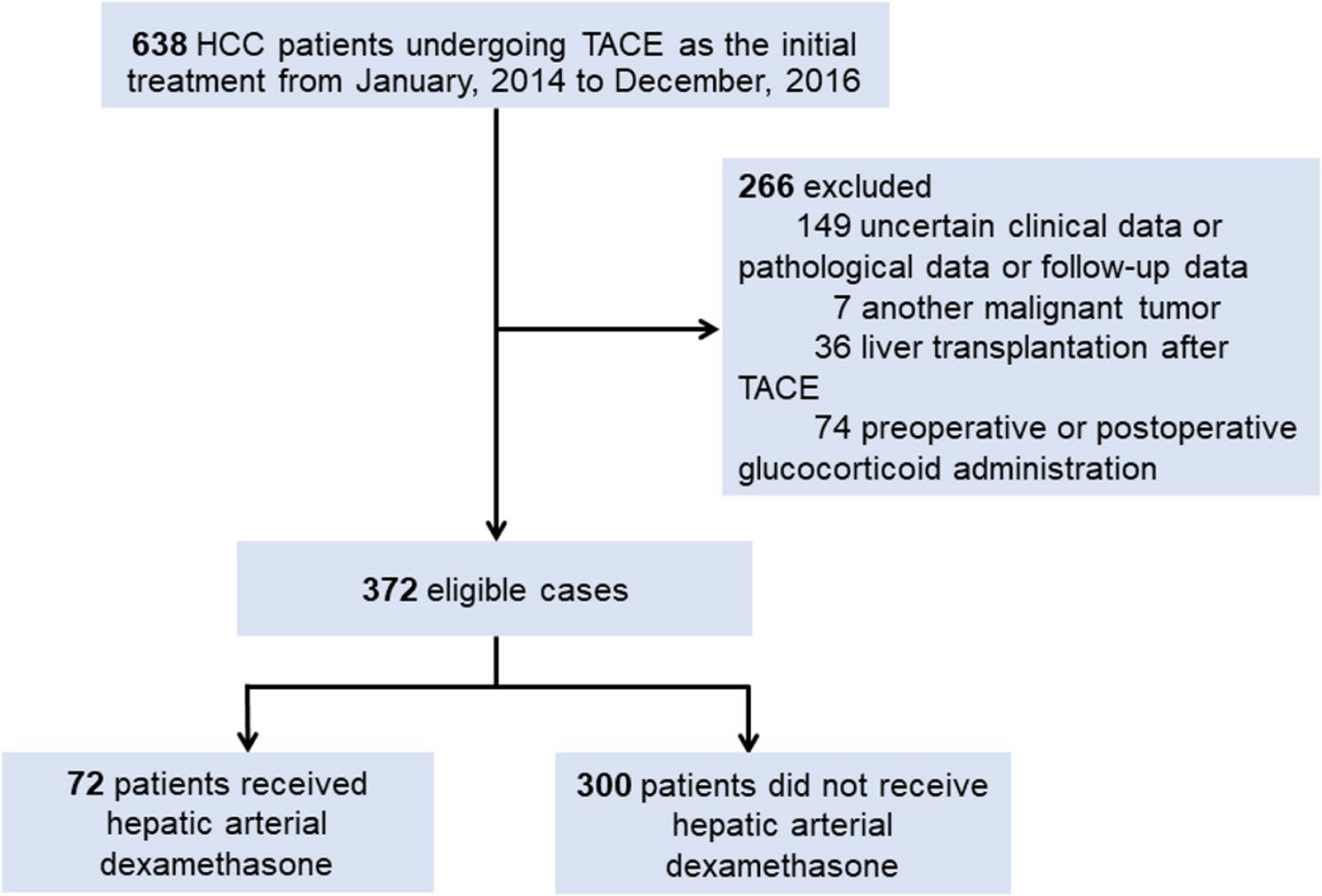
Flow diagram of study patient selection

The exclusion criteria included: preoperative or postoperative administration of glucocorticoid, another malignant tumor, subsequent transplantation, incomplete clinical data, uncertain pathological data and unknown follow-up data (Fig. 1).

All data were extracted from the SYSUCC electronic medical record, encompassing a range of variables: age, sex, tumor number, tumor size, metastasis, Barcelona Clinic Liver Cancer stage (BCLC) stage, portal vein tumor thrombus (PVTT), hepatitis B virus level, albumin level, total bilirubin level, alpha fetoprotein level, and chemotherapy protocol and adjuvant medications for TACE.

### TACE and hepatic arterial dexamethasone

We conducted TACE using the technique as previously described [16]. After visceral angiography, a microcatheter was used to catheterize super-selectively into the vessels suppling the tumors. Chemolipiodolization was carried out using carboplatin (300 mg) or lobaplatin (50 mg) or no platinum, followed by a mixture of epirubicin 50 mg and lipiodol 2-5 ml. Then, embolization was performed by injecting polyvinyl alcohol particles. Subsequently, the extent of embolism was confirmed by angiography. TACE was repeated once every 6 weeks. For all cases, the procedure was performed by physicians who had a more than 5 years of experience in interventional therapy for hepatocellular carcinoma. During the TACE procedure, dexamethasone is administered via the hepatic artery before the infusion of the chemotherapeutic agents to prevent post-embolization syndrome (PES) resulting from subsequent chemotherapy and embolization.

### Patient follow-up and clinical outcomes

HCC patients were followed at regular intervals after TACE, either through clinic visits or hospital stays, to monitor their AFP level, liver function, complete blood count, coagulation parameters and abdominal CT/MR.

Our primary outcome was progression-free survival. Progression-free survival was defined as the time in months from the date of first TACE to the date of radiographic tumor progression or death [17]. The secondary outcome, overall survival (OS), was defined as the time (in months) from the first TACE procedure until death or the last follow-up. Both primary and secondary outcomes were specified before statistical analysis. The follow-up period extended until April 30, 2025.

### Statistical analysis

As a retrospective analysis, the sample size was determined based upon the available data from SYSUCC. Continuous variables were presented as mean ± standard deviation (SD), if it conformed to a normal distribution; otherwise, they were presented as median (inter-quartile range). Categorical variables were described as number (percentage). Continuous variables were compared using the Student’s t test or Kruskal–Wallis test, depending on their distribution. Categorical variables were compared using the Chi-square test or Fisher’s exact test, as appropriate. An initial univariable and multivariable cox proportional hazards regression analyses were undertaken to assess the association between hepatic arterial dexamethasone administration during TACE and survival outcomes in the overall population. In addition, we performed propensity score methods to mitigate the effects of confounding variables. The individual propensities for hepatic arterial dexamethasone administration were calculated using multivariable logistic regression model. Covariates for multivariable cox regression analyses and propensity score methods were chosen based on clinical significance, existing research and initially imbalanced variables. The following covariates were used: age, sex, HBV, ALB level, TBIL level, AFP level, tumor number, tumor size, metastasis, BCLC stage and PVTT. The primary analysis used inverse probability of treatment weight (IPTW), in which the stabilized inverse-probability-weighting weights were derived using the predicted probabilities from the propensity-score model. Propensity score matching was also performed. We chose weighted full matching to achieve adequate balance, and retain all treated and control units [18]. Standardized mean differences (SMDs) were used to assess the quality of matching. Additionally, we conducted another analysis that included the propensity score as an additional covariate. Kaplan–Meier analysis was performed to estimate survival differences between two groups, and the log-rank test was used to assess statistical significance. Results were shown as hazard ratios (HRs) with 95% confidence intervals (95% CIs). Subgroup analyses were performed to evaluate the association between hepatic arterial dexamethasone and survival outcomes in pre-specified subgroups. All statistical analyses were performed using R (version 4.2.0, R Foundation for Statistical Computing, Vienna, Austria) and IBM SPSS Statistics (version 22.0). *P* values less than 0.05 were considered statistically significant.

## Results

**Fig. 2:**
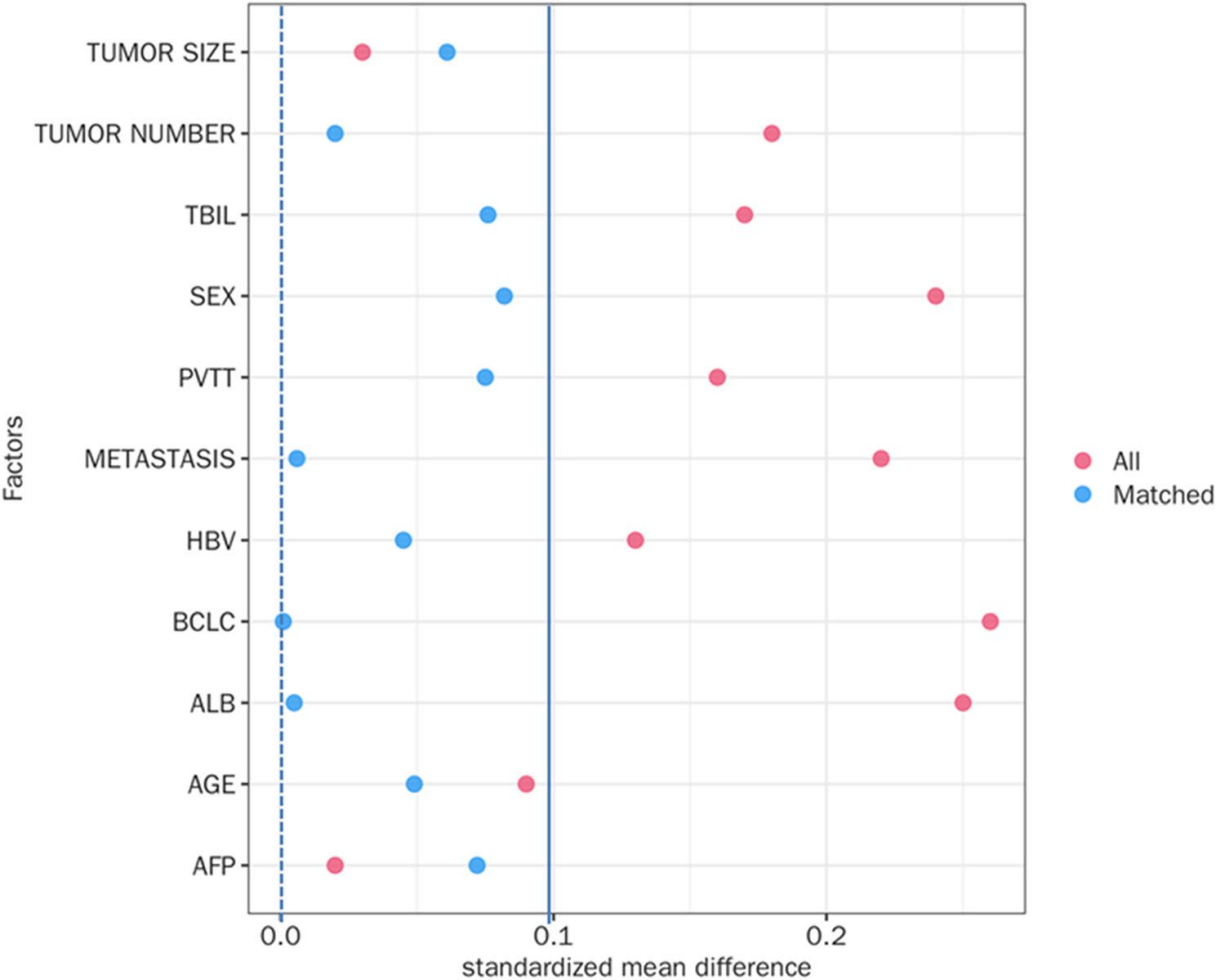
Assessing the quality of matching

We identified 638 HCC patients undergoing TACE as first-line therapy between January 2014 and December 2016 in the SYSUCC database. 372 patients were included in the subsequent analyses, among which 72 patients received hepatic arterial dexamethasone (dexamethasone group) and 300 patients did not receive hepatic arterial dexamethasone (no dexamethasone group). The study flowchart is presented in Fig. 1. As summarized in Table S1, significant differences between two groups were observed in the following baseline characteristics: sex, HBV, ALB, TBIL, tumor number, metastasis, BCLC stage and PVTT. To balance baseline differences and minimize the impact of confounding factors, three propensity-score methods were performed. After full matching on the propensity score, 72 patients were assigned to the dexamethasone group and 300 patients to the no dexamethasone group. Balance after full matching between the two groups was successfully achieved for all variables (SMD < 0.1; Table 1). The SMDs for all covariates before and after full matching are illustrated in Fig. 2. In addition, HCC patients were treated with a median of 2 cycles of TACE (range:1–6). Among patients who received hepatic arterial dexamethasone during TACE, the drug was administered via a fixed-dose regimen, with a median dose of 8 mg (range: 5–10 mg). The dose distribution was 5 mg in 11 patients, 8 mg in 47 patients, and 10 mg in 14 patients.

**Table 1:** Characteristics of patients undergoing TACE in the dexamethasone and no dexamethasone groups after propensity score matching and inverse probability weighting

Variables	No dexamethasone (*N* = 300)	Dexamethasone (*N* = 72)	SMD
	*N* (W%)	*N* (W%)	
Age (year)			0.049
≤ 50	100(31.4%)	21(29.2%)	
> 50	200(68.6%)	51(70.8%)	
Sex			0.082
Male	277(81.7%)	61(84.7%)	
Female	23(18.3%)	11(15.3%)	
HBV			0.045
Absent	27(6.6%)	4(5.6%)	
Present	273(93.4%)	68(94.4%)	
ALB (g/L)			0.005
≤ 40	149(37.7%)	27(37.5%)	
> 40	151(62.3%)	45(62.5%)	
TBIL (µmol/L)			0.076
≤ 20	220(77.5%)	58(80.6%)	
> 20	80(22.6%)	14(19.4%)	
AFP (ng/mL)			0.072
≤ 200	135(42.3%)	33(45.8%)	
> 200	165 (57.8%)	39(54.2%)	
Tumor number			0.020
≤ 3	135 (55.2%)	39(54.2%)	
> 3	165(44.8%)	33(45.8%)	
Tumor size (cm)			0.061
≤ 10	209(65.2%)	49(68.1%)	
> 10	91(34.8%)	23(31.9%)	
Metastasis			0.006
Absent	271(95.7%)	69(95.8%)	
Present	29(4.3%)	3(4.2%)	
BCLC stage			0.001
B	194(76.4%)	55(76.4%)	
C	106(23.6%)	17(23.6%)	
PVTT			0.075
Absent	212(80.8%)	56(77.8%)	
Present	88(19.2%)	16(22.2%)	

*AFP *alpha fetoprotein, *ALB *albumin, *BCLC *Barcelona Clinical Liver Cancer, *HBV *hepatitis B virus, *PSM *propensity score matching, *PVTT *portal vein tumor thrombus, *SMD *standardized mean difference, *TACE *transcatheter arterial chemoembolization, *TBIL *total bilirubin, *W% *weighted percentage

### Primary outcome

**Fig. 3:**
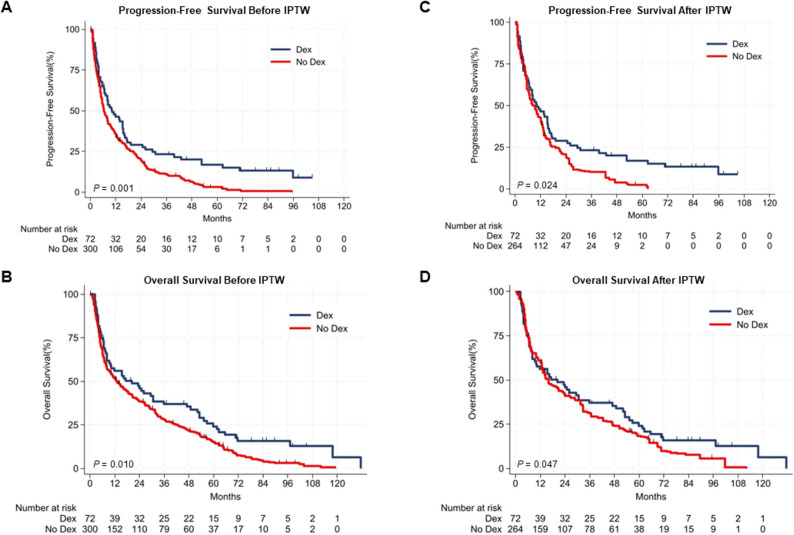
Kaplan–Meier progression-free survival and overall survival curves between the dexamethasone group and no dexamethasone group. The shaded areas indicate 95% confidence intervals. Blue line shows dexamethasone group. Red line shows no dexamethasone group. **A** Progression-free survival before IPTW; (**B**) Overall survival before IPTW; (**C**) Progression-free survival after IPTW; (**D**) Overall survival after IPTW. HCC, hepatocellular carcinoma; IPTW, inverse probability of treatment weight; TACE, transcatheter arterial chemoembolization

Amongst the 372 patients, the progression rate was 84.7% (61/72) in the dexamethasone group and 95.0% (285/300) in the no dexamethasone group, respectively. The median PFS in the dexamethasone and no dexamethasone group were 10.20 months vs. 6.43 months, and PFS differed significantly between the two groups in the unadjusted analysis (HR = 0.61; 95%CI, 0.46–0.82; *P* = 0.001; Fig. 3A and Table 2). Multivariable cox proportional-hazards regression showed that hepatic arterial dexamethasone remained significantly associated with prolonged PFS. (HR = 0.67; 95%CI, 0.50–0.90; *P* = 0.008) (Table 2). After full matching on the propensity score, we found that patients who received hepatic arterial dexamethasone were more likely to have better progression-free survival (HR = 0.51; 95%CI, 0.33–0.78; *P* = 0.002). In the inverse probability weighting according to the propensity score, a significant association was observed between hepatic arterial dexamethasone and prolonged PFS (HR = 0.69; 95%CI, 0.49–0.95; *P* = 0.024; Fig. 3C and Table 2). Additional propensity-score analysis, which was adjusted for propensity score, showed consistent results (HR = 0.68; 95%CI, 0.51–0.91; *P* = 0.009) (Table 2).

**Table 2:** Associations between intra-arterial dexamethasone administration and survival outcomes in the crude analysis, multivariable analysis, and propensity-score analyses

Analysis	PFS	*P* - value	OS	*P* - value
Median survival time (months)				
Dexamethasone group	10.20		20.40	
Control group	6.43		12.90	
Crude analysis – hazard ratio (95%CI)	0.61(0.46–0.82)	0.001	0.69(0.52–0.91)	0.010
Multivariable analysis – hazard ratio (95%CI)	0.67(0.50–0.90)	0.008	0.72(0.54–0.96)	0.027
Propensity-score analyses – hazard ratio (95%CI)				
With matching	0.51(0.33–0.78)	0.002	0.60(0.41–0.90)	0.014
With inverse probability weighting	0.69(0.49–0.95)	0.024	0.72(0.52-1.00)	0.047
Adjusted for propensity score	0.68(0.51–0.91)	0.009	0.75(0.56-1.00)	0.049

Propensity scores were calculated using logistic regression according to age, sex, HBV, ALB, TBIL, AFP, tumor number, tumor size, metastasis, BCLC stage, and PVTT

*AFP *alpha fetoprotein, *ALB *albumin, *BCLC *Barcelona Clinical Liver Cancer, *CI *confidence interval, *HBV *hepatitis B virus, *HR *hazard ratio, *IQR *interquartile range, *OS *overall survival, *PFS *progression-free survival, *PSM *propensity score matching, *PVTT *portal vein tumor thrombus, *TACE *transcatheter arterial chemoembolization, *TBIL *total bilirubin

### Secondary outcome

In the overall population, the mortality in the dexamethasone and no dexamethasone group were 83.3% (60/72) vs. 91.7% (275/300). The dexamethasone group had a median OS of 20.40 months, and 12.90 months in the no dexamethasone group. The unadjusted analysis showed statistically significant association between hepatic arterial dexamethasone and improved OS (HR = 0.69; 95%CI, 0.52–0.91; *P* = 0.010; Fig. 3B and Table 2). In the multivariable cox regression analysis, we also observed that hepatic arterial dexamethasone during TACE was correlated with better OS in HCC patients (HR = 0.72; 95%CI, 0.54–0.96; *P* = 0.027) (Table 2). The results of PSM was in agreement with those of the crude and multivariable regression analysis. (HR = 0.60; 95%CI, 0.41–0.90; *P* = 0.014). Consistent association was also found in the inverse probability weighting analysis (HR = 0.72; 95%CI, 0.52-1.00; *P* = 0.047; Fig. 3D and Table 2) and additional propensity-score analysis (HR = 0.75; 95%CI, 0.56-1.00; *P* = 0.049) (Table 2).

### Subgroup analysis

Since dexamethasone is commonly co-administered with chemotherapy such as platinum, how dexamethasone affects the effect of platinum may warrant more attentions in clinical practice. Previous study reported that dexamethasone might increase the chemosensitivity of HCC cells to cisplatin, so we performed subgroup analyses to determine the association between hepatic arterial dexamethasone administration during TACE and survival outcomes in HCC patients receiving platinum-based or non-platinum-based TACE. We found that hepatic arterial dexamethasone decreased risk of cancer progression (HR = 0.51; 95%CI, 0.31–0.84; *P* = 0.007) and death (HR = 0.63; 95%CI, 0.46–0.87; *P* = 0.004) in HCC patients undergoing TACE with platinum (Figure S1A and S1C). Additionally, for dexamethasone group compared with no dexamethasone group, we observed an HR of 0.83 (95%CI, 0.37–1.85; *P* = 0.650) for PFS and an HR of 0.73 (95%CI, 0.37–1.45; *P* = 0.362) for OS in HCC patients undergoing TACE without platinum (Figure S1B and S1D). Despite this, the effect of hepatic arterial dexamethasone administration on PFS and OS was consistent across all the other subgroups, irrespective of age, sex, HBV infection status, ALB level, TBIL level, AFP level, tumor number, tumor size, metastasis status, BCLC stage and PVTT status (all *P* interaction > 0.05; Figure S2).

In order to eliminate the impact of subsequent treatment strategies, we also collected and analyzed all post-TACE therapies, including conversion resection, ablation, and systemic treatments. No statistically significant differences were observed between the two groups in the distribution of post-TACE therapies (Table S2).

## Discussion

In this retrospective cohort study, a significant association was identified between hepatic arterial dexamethasone and improved long-term survival in HCC patients who received TACE as first-line therapy. Additionally, this association was similar with the findings in subgroup patients receiving TACE with platinum. This study not only attempted to minimize the confounding effect by utilizing three propensity score methods, but also found a consistent impact of hepatic arterial dexamethasone administration on survival outcomes among HCC patients undergoing TACE. Our study highlights the clinical significance of hepatic arterial dexamethasone administration in HCC patients undergoing TACE.

We speculate that several factors could account for the long-term survival benefit of hepatic arterial dexamethasone during TACE. First, dexamethasone has anti-inflammatory property. It is now becoming clear that anti-inflammatory agents may be a good option for cancer prevention and therapy [19]. Dexamethasone could attenuate tumor progression via suppressing secretion of pro-inflammatory cytokines, activation of inflammatory cells and macrophage polarization-mediated inflammation [20, 21]. Second, dexamethasone could inhibit tumor angiogenesis. Previous studies indicated that dexamethasone suppressed tumor-associated angiogenesis through glucocorticoid receptor by regulating vascular endothelial growth factor, COX-2 and interleukin-8 [22, 23]. Also, laboratory evidence suggested that dexamethasone could impact glucose metabolism of HCC cells by augmenting the gluconeogenesis pathway, thus inhibiting the growth of cancer cells [24]. Besides, dexamethasone may inhibit the maintenance of stemness in hepatocellular carcinoma stem cells. Recent studies have demonstrated that dexamethasone concurrently reduced hypoxia tolerance and malignant phenotype of stem cells while inhibiting epithelial-to-mesenchymal transition and migration by inducing deSUMOylation of Oct4 and HIF-1α [13]. However, dexamethasone per se is not strong enough to eradicate cancer cells, it must synergize with chemotherapeutics. Increasing evidences showed that dexamethasone might enhance the antitumor efficacy of some chemotherapeutic agents. Dexamethasone sensitized HCC cells to cisplatin by synchronizing cancer cells at the G2/M phase, because G2/M synchronized cells were more vulnerable to cisplatin-induced cell death [12]. A similar result reported that dexamethasone increased the sensitivity of cancer cells to Gemcitabine in HCC, breast carcinoma and ovarian cancer [25, 26] Aforementioned possible reasons might interpret the clinically meaningful improvements of hepatic arterial dexamethasone administration observed in our study.

The effect of dexamethasone on oncological outcomes were divergent in a variety of tumors. For example, in ovarian cancer patients after cytoreductive surgery, intraoperative dexamethasone had no impact on long-term survival [27]. The results were similar to non-small cell lung cancer patients [28]. Also, rectal cancer patients who received dexamethasone had worse three-year DFS and OS after curative surgery [29]. A more interesting thing was that intraoperative dexamethasone administration could provide long-term survival benefit in pancreatic adenocarcinoma patients [30, 31]. Besides, a large sample size cohort study showed that dexamethasone was associated with lower 1-year mortality and improved recurrence-free survival in non-immunotherapy patients undergoing resection surgery of cancers [32]. A meta-analysis, which included 7066 patients from 15 prospective and retrospective studies, suggested that perioperative glucocorticoids improved DFS and 1-year mortality [33]. These distinct and even contradictory results could imply a cell-type specific effect of dexamethasone. Another possible explanation might be the differential expression of GR in diverse cell types [34]. Previous study demonstrated that the effect of DEX on the growth and chemosensitivity of cancer cells was closely linked to the level of GR content [35]. However, the exact mechanism for this differential effect of DEX remains unknown until now. 

Our finding was reminiscent of the randomized trial reported by Nancy et al. showing that hepatic arterial dexamethasone infusion was not associated with better survival in hepatic metastases patients [15]. However, the following several respects in the current study differed distinctly from this randomized trial. Our study population was limited to hepatocellular carcinoma patients, while the population of this randomized trial was patients with liver metastases from colorectal carcinoma. Compared with HCC patients who received TACE as first-line therapy in our study, metastatic colorectal cancer patients in the randomized trial might be heavily treated preoperatively. The potential effects of dexamethasone on long-term oncological outcomes could have been masked by complicated preoperative therapy. Dosage, injection timing, route and frequency for dexamethasone administration were also different between these two studies, which might play a decisive role in different oncological outcomes. Moreover, Nancy et al. reported that fluorodeoxyuridine (FUDR) plus intrahepatic dexamethasone was associated with an increased response rate in hepatic metastasis, we found a survival benefit in HCC patients receiving platinum and hepatic arterial dexamethasone. As stated in aforementioned studies, dexamethasone could sensitize HCC cells to platinum-based chemotherapy. This might be an important reason why we observed a clinical meaningful improvement in our study.

We acknowledge that this study entailed several limitations. We couldn’t completely avoid selection bias, because it was retrospective in nature. In order to minimize the possible confounding effect of each variable, strict inclusion and exclusion criteria were applied and propensity score methods were performed. This study had a relatively small sample size. To mitigate the instability of results caused by limited sample size, we carried out three propensity score methods and observed a consistent result. We also conducted full matching on the propensity score to retain more treated and control units. Moreover, no standardized guidelines or uniformly validated criteria exist to identify patients at high risk for postembolization syndrome following transcatheter arterial chemoembolization. In our clinical practice, we identified patients at increased risk for postembolization syndrome by integrating limited available evidence (e.g., large tumor burden, history of prior postembolization syndrome) with clinical experience. Despite this, considering the validity of propensity score methods and outstanding statistical significance of hepatic arterial dexamethasone observed in our study, these identified limitations could not alter the conclusions.

## Conclusion

We found that hepatic arterial dexamethasone administration was associated with improved PFS and OS in HCC patients undergoing TACE as the initial treatment. The results were consistent in subgroup patients receiving TACE with platinum. Our study presents novel evidence indicating that hepatic arterial dexamethasone administration during TACE may provide substantial survival benefit in HCC patients. Well-designed prospective studies are necessary to further validate these observed associations.

## Supplementary Information

Supplementary Material 1.

Supplementary Material 2.

## Data Availability

Some or all datasets generated during and/or analyzed during the current study are not publicly available but are available from the corresponding author on reasonable request.
