# Supplementary material for: Effect of hepatic arterial dexamethasone administration on long-term survival among hepatocellular carcinoma patients undergoing transcatheter arterial chemoembolization
Source: BMC Anesthesiol. 2026 Jun 25;26:448. doi: 10.1186/s12871-026-04054-w (PMC13401781; doi:10.1186/s12871-026-04054-w)
Supplement: Supplementary file 1 — Supplementary Material 1. [file 12871_2026_4054_MOESM1_ESM.docx]

**Table S1 Characteristics of patients undergoing TACE in the dexamethasone and no dexamethasone groups before propensity score matching and inverse probability weighting.**

| Variables | No dexamethasone (N=300) | Dexamethasone (N=72) | SMD |
| --- | --- | --- | --- |
|  | N (W%) | N (W%) |  |
| Age (year) |  |  | 0.090 |
| ≤50 | 100(33.3%) | 21(29.2%) |  |
| >50 | 200(66.7%) | 51(70.8%) |  |
| Sex |  |  | 0.241 |
| Male | 277(92.3%) | 61(84.7%) |  |
| Female | 23(7.7%) | 11(15.3%) |  |
| HBV |  |  | 0.133 |
| Absent | 27(9.0%) | 4(5.6%) |  |
| Present | 273(91.0%) | 68(94.4%) |  |
| ALB (g/L) |  |  | 0.247 |
| ≤40 | 149(49.7%) | 27(37.5%) |  |
| >40 | 151(50.3%) | 45(62.5%) |  |
| TBIL (μmol/L) |  |  | 0.172 |
| ≤20 | 220(73.3%) | 58(80.6%) |  |
| >20 | 80(26.7%) | 14(19.4%) |  |
| AFP (ng/mL) |  |  | 0.017 |
| ≤200 | 135(45.0%) | 33(45.8%) |  |
| >200 | 165 (55.0%) | 39(54.2%) |  |
| Tumor number |  |  | 0.184 |
| ≤3 | 135 (45.0%) | 39(54.2%) |  |
| >3 | 165(55.0%) | 33(45.8%) |  |
| Tumor size (cm) |  |  | 0.035 |
| ≤10 | 209(69.7%) | 49(68.1%) |  |
| >10 | 91(30.3%) | 23(31.9%) |  |
| Metastasis |  |  | 0.218 |
| Absent | 271(90.3%) | 69(95.8%) |  |
| Present | 29(9.7%) | 3(4.2%) |  |
| BCLC stage |  |  | 0.259 |
| B | 194(64.7%) | 55(76.4%) |  |
| C | 106(35.3%) | 17(23.6%) |  |
| PVTT |  |  | 0.163 |
| Absent | 212(70.7%) | 56(77.8%) |  |
| Present | 88(29.3%) | 16(22.2%) |  |

AFP, alpha fetoprotein; ALB, albumin; BCLC, Barcelona Clinical Liver Cancer; HBV, hepatitis B virus; PSM, propensity score matching; PVTT, portal vein tumor thrombus; SMD, standardized mean difference; TACE, transcatheter arterial chemoembolization; TBIL, total bilirubin; W%, weighted percentage.

**Table S2 Post-TACE treatments stratified by hepatic arterial dexamethasone administration during transcatheter arterial chemoembolization. Data are presented as n (%). * P-values were calculated using a two-sided χ2 test.**

| Variable |  | Hepatic arterial dexamethasone administration | | | |
| --- | --- | --- | --- | --- | --- |
|  |  | No (N=300) | Yes (N=72) | P value* | SMD |
| Post-TACE treatments |  |  |  | 0.224 | -0.08 |
| None |  | 24(8.0%) | 8(11.1%) |  |  |
| Ablation |  | 100(33.3%) | 20(27.8%) |  |  |
| Conversion resection |  | 41(13.7%) | 5(6.9%) |  |  |
| Systemic therapy |  | 135(45.0%) | 39(54.2%) |  |  |
