# Supplementary material for: Effect of hepatic arterial dexamethasone administration on long-term survival among hepatocellular carcinoma patients undergoing transcatheter arterial chemoembolization
Source: BMC Anesthesiol. 2026 Jun 25;26:448. doi: 10.1186/s12871-026-04054-w (PMC13401781; doi:10.1186/s12871-026-04054-w)

**Figure S1. Kaplan–Meier progression-free survival and overall survival curves for subgroup patients stratified by hepatic arterial dexamethasone. The shaded areas indicate 95% confidence intervals. Blue line shows dexamethasone group. Red line shows no dexamethasone group. A: Progression-free survival in patients receiving TACE with platinum; B: Overall survival in patients receiving TACE with platinum; C: Progression-free survival in patients receiving TACE without platinum; D: Overall survival in patients receiving TACE without platinum. TACE, transcatheter arterial chemoembolization.**


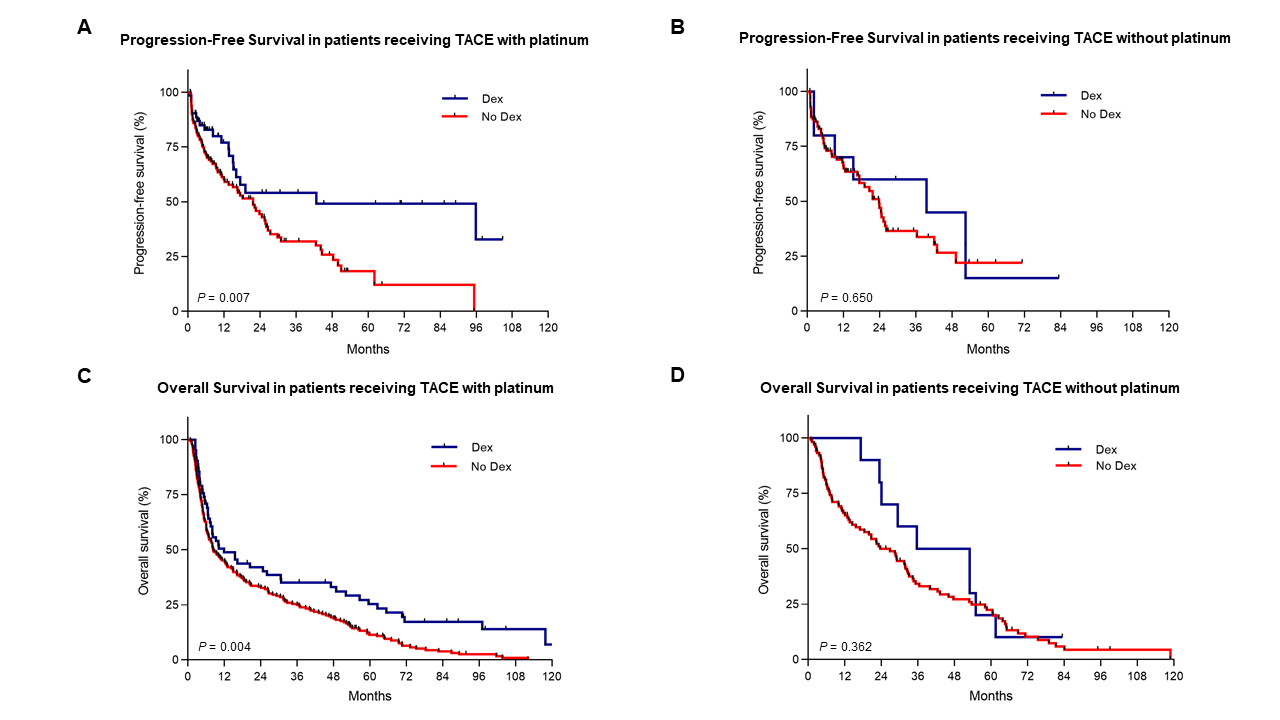


**Figure S2. Stratified associations between intra-arterial dexamethasone administration and survival outcomes by age, sex, HBV, ALB, TBIL, AFP, tumor number, tumor size, metastasis, BCLC stage and PVTT. A: Progression-free survival; B: Overall survival. AFP, alpha fetoprotein; ALB, albumin; BCLC, Barcelona Clinical Liver Cancer; HBV, hepatitis B virus; PVTT, portal vein tumor thrombus; TBIL, total bilirubin.**


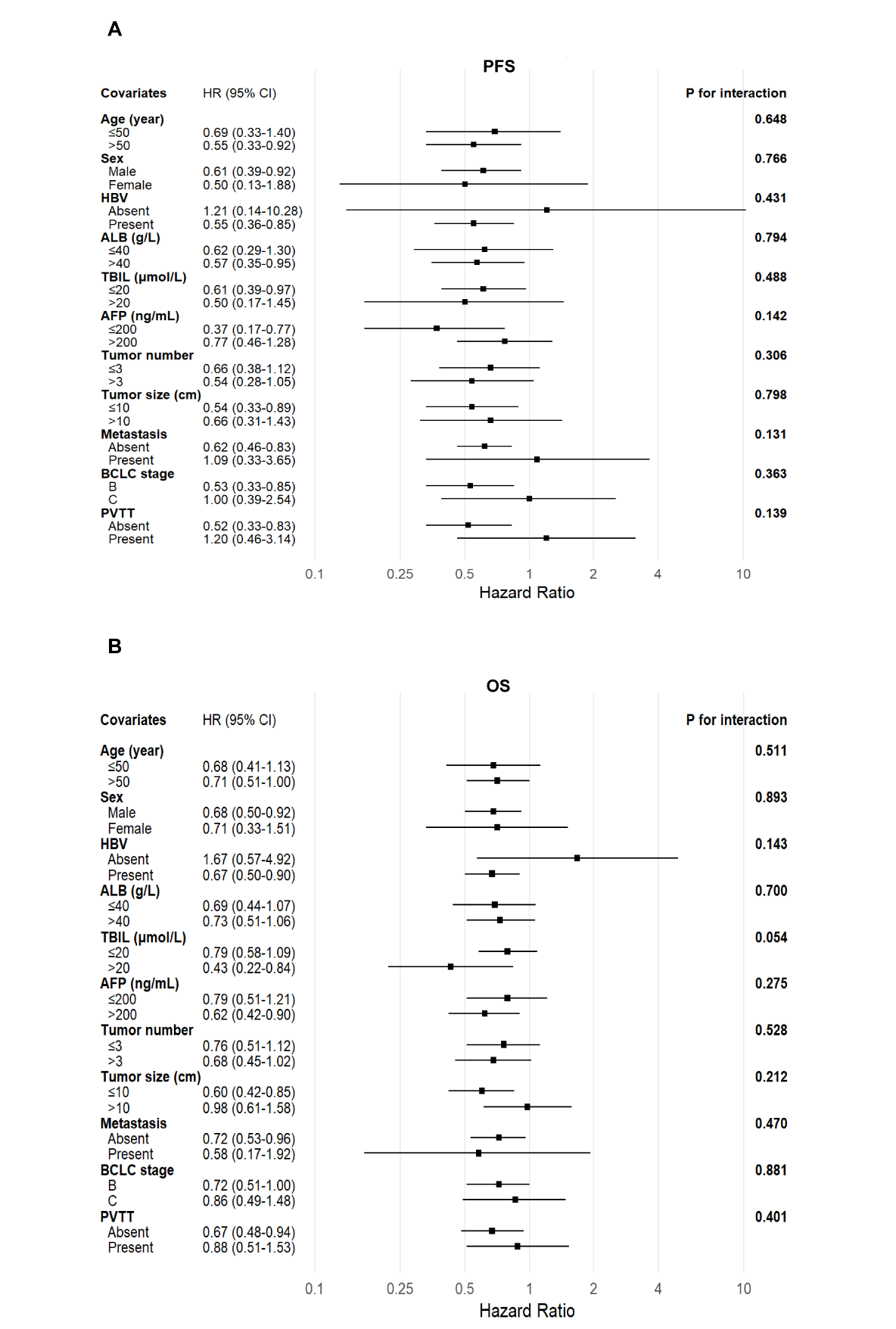

Supplement: Supplementary file 2 — Supplementary Material 2. [file 12871_2026_4054_MOESM2_ESM.docx]
